# Effects of Exercise and Milk Fat Globule Membrane (MFGM) Supplementation on Body Composition, Physical Function, and Hematological Parameters in Community-Dwelling Frail Japanese Women: A Randomized Double Blind, Placebo-Controlled, Follow-Up Trial

**DOI:** 10.1371/journal.pone.0116256

**Published:** 2015-02-06

**Authors:** Hunkyung Kim, Takao Suzuki, Miji Kim, Narumi Kojima, Noriyasu Ota, Akira Shimotoyodome, Tadashi Hase, Erika Hosoi, Hideyo Yoshida

**Affiliations:** 1 Research Team for Promoting Independence of the Elderly, Tokyo Metropolitan Institute of Gerontology, Tokyo, Japan; 2 National Institute for Longevity Sciences, Aichi, Japan; 3 Biological Science Laboratories, Health Science, Kao Corporation, Tochigi, Japan; Vanderbilt University, UNITED STATES

## Abstract

**Objective:**

To investigate the combined and separate effects of exercise and milk fat globule membrane (MFGM) supplementation on frailty, physical function, physical activity level, and hematological parameters in community-dwelling elderly Japanese women.

**Methods:**

A total of 131 frail, elderly women over 75 years were randomly assigned to one of four groups: exercise and MFGM supplementation (Ex+MFGM), exercise and placebo (Ex+Plac), MFGM supplementation, or the placebo group. The exercise group attended a 60-minute training program twice a week for three months, and the MFGM group ingested 1g of the MFGM supplement in pill form, daily for 3 months. The primary outcome measure was change in frailty status based on Fried’s frailty phenotype. Secondary outcome measures included body composition, physical function and hematological parameters, and interview survey components assessing lifestyle factors. Participants were followed for 4 months post-intervention.

**Results:**

Significant group×time interactions were observed for usual walking speed (P = 0.005), timed up & go (P<0.001), and insulin-like growth factor-binding protein 3 / insulin-like growth factor 1 ratio (P = 0.013). The frailty components revealed that weight loss, exhaustion, low physical activity, and slow walking speed were reversed, but low muscle strength did not significantly changed. Frailty reversal rate was significantly higher in the Ex+MFGM (57.6%) than in the MFGM (28.1%) or placebo (30.3%) groups at post-intervention (χ^2^ = 8.827, P = 0.032), and at the follow-up was also significantly greater in the Ex+MFGM (45.5%) and Ex+Plac (39.4%) groups compared with the placebo (15.2%) group (χ^2^ = 8.607, P = 0.035). The exercise+MFGM group had the highest odds ratio (OR) for frailty reversal at post-intervention and follow-up (OR = 3.12, 95% confidence interval (CI) = 1.13–8.60; and OR = 4.67, 95% CI = 1.45–15.08, respectively).

**Conclusion:**

This study suggests that interventions including exercise and nutrition can improve frailty status. Statistically significant additive effects of MFGM with exercise could not be confirmed in this population, and further investigation in larger samples is necessary.

**Trial Registration:**

The Japan Medical Association Clinical Trial Registry (JMACCT)JMA-IIA00069

## Introduction

Frailty has been a focus in the aging literature in the past decade. There are several risk factors related to frailty such as aging, chronic disease, skeletal muscle disuse and cognitive function decline, although the mechanisms are still unclear [1,2]. Regardless, presence of these risk factors may lead to declines in physiological function, fractures, activities of daily living (ADL) disabilities, hospitalization, and death [3,4]. There is; however, still a lack of consensus on the assessment and definition of frailty. One of the most commonly used definitions based on phenotypic classification, defines frailty as the presence of three or more of five components which includes weight loss, muscle weakness, exhaustion, slow walking speed, and low physical activity level [3].

Exercise has been a focus in the prevention of frailty, as research has shown that it is beneficial for the enhancement of skeletal muscle mass and strength, and can improve muscle function, physical activity participants and functional ability in frail older adults [5]. Whether or not exercise can reverse frailty status, as an outcome measure, still remains to be confirmed. There has also been an increase in interest regarding the value of nutritional supplementation for skeletal muscle enhancement. Several studies have investigated the effects of milk ingestion on body composition and protein synthesis as well as muscle mass [6–8]. The results of these studies showed that the consumption of milk with resistance training may have increased muscle protein synthesis thus promoting muscle mass maintenance and gains, and even increased strength gains. However, these studies were performed on young adults, and the results of milk consumption with exercise on the elderly population are unknown. Recently, the nutritional properties of milk fat globule membrane (MFGM) have been under study. MFGM is a highly complex structure made from different protein and lipid components of milk, and its biological importance has been under study, as it is considered a potentially valuable ingredient for new food products [9]. Although the research on MFGM is still in its preliminary stages, one mice study found that MFGM in congruence with habitual exercise effectively suppressed the aging-associated deterioration of muscle mass and strength in aging mice[10]. However, the effects of MFGM supplementation alone and combined with exercise in frail elderly people, remains to be confirmed.

Therefore the purpose of this study was to investigate the effects of the combined and separate effects of exercise and MFGM supplementation on frailty reversal, physical function, and hematological parameters in community-dwelling elderly Japanese women.

## Methods

The protocol for this trial and supporting CONSORT checklist are available as supporting information; see S1 CONSORT Checklist and S1 Protocol.

### Ethics Statement

We conducted a randomized double-blind placebo-controlled trial. The study protocol was approved by the Clinical Research Ethics Committee of the Tokyo Metropolitan Institute of Gerontology (TMIG). The intervention procedures were fully explained to all participants and written informed consents were obtained. The study was registered at The Japan Medical Association Clinical Trial Registry (JMACCT) JMA-IIA00069.　The authors confirm that all ongoing and related trials for this intervention are registered. Although the date of the registration is stated as August 11, 2011 (after recruitment began for the trial), the process was started on June 24, 2011 prior to recruitment. The registration took longer than anticipated because we were unfamiliar with the system and the process, and this led to the delayed registration date.

### Study Population and Timeline

An invitation letter to participate in an annual comprehensive general health survey was mailed to elderly women who were randomly selected from the Basic Resident Register of elderly people residing in the Itabashi ward of Tokyo Japan. The invitation was sent to a cohort of 1,447 women aged 77 and older in November 2009, where 974 people participated in the health survey. In October 2010, the invitation was sent to a different cohort of 1,458 people aged 74 and older, and 861 people volunteered for the survey. A total of 1,835 people participated in the baseline assessment at the TMIG, however the 1,070 who were invited but did not participate could not be followed.　

The Clinical Research Ethics Committee of the TMIG approved this study on May 26, 2011. The recruitment period was from July 1∼July 13, 2011 and the information session for potential participants was held on July 28, 2011. Volunteer participation forms were collected from people who were interested in the study, and they were asked to read through the informed consent form at home. The baseline survey was held across two days from August 30∼August 31, 2011, and the signed informed consent forms were obtained on these days. Randomization was performed by the principal investigator between September 4∼September 5, 2011, and the group assignments were sent to the participants on September 8, 2011. The first phase of the intervention began on September 14, 2011, until the post-intervention survey on December 5∼December 6, 2011. Participants were followed for four months, and the follow-up survey was held on March 14∼March 15, 2012. The groups were then crossed over for the second phase of the study, where the intervention began on March 26, 2012. The post-intervention survey for the second phase was held on June 13∼June 14, 2012, marking the end of this trial. For the scope of this study, the data from the cross-over was not analyzed. The data analyzed in this study only included the first phase of the trial, which was until the follow-up (March 14∼March 15, 2012).

### Inclusion and Exclusion Criteria for Intervention

Participation in the intervention required the participants to be operationally defined as frail, which meant categorization into three or more of the following inclusion criteria [3]: 1) Weight loss: At baseline, having unintentionally lost more than 2.0∼3.0kg in the last 6 months; since the intervention was 3 months, i.e., half of the 6 months, the weight loss criteria post-intervention was 1.0∼1.5kg. The follow-up period was 4 months, or two-thirds of the 6 months, the weight loss criteria was two-thirds of the original, 1.3∼2.0kg at follow-up. 2) Weakness: grip strength less than 19.0kg; 3) Slow walking speed: usual walking speed less than 1.0 m/s; 4) Exhaustion: Answering “yes” to at least one of two questions, “I felt that everything I did was an effort” or “I could not get going”; 5) Low activity: Answering “true” to at least 3 of the following 4 statements, “I regularly take walks less than once a week,” “I do not exercise regularly,” “I do not actively participate in hobbies or lessons of any sort,” and “I do not participate in any social groups for elderly people or volunteering.”

**Fig 1:**
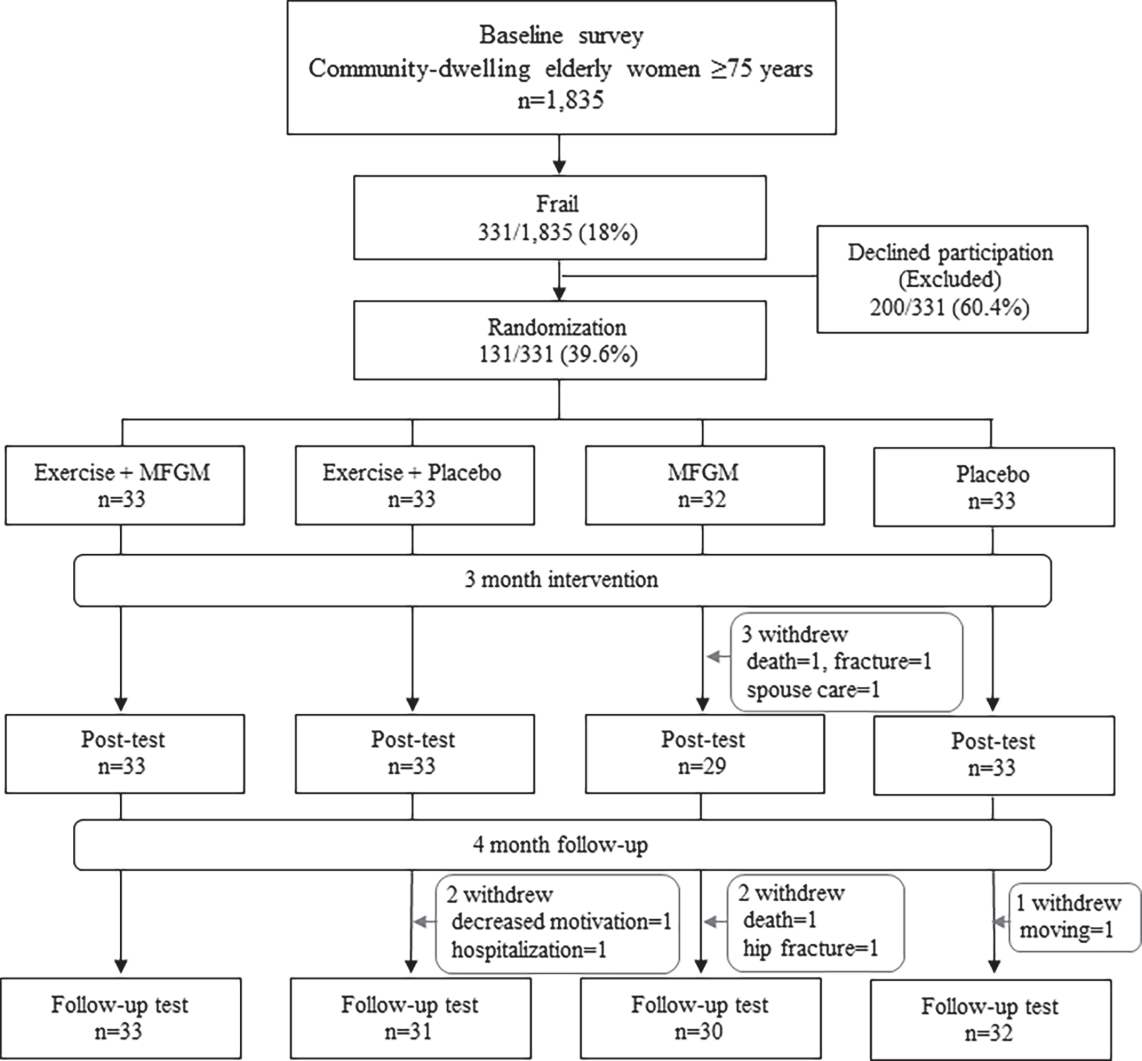
Flow chart of participant recruitment during the randomized controlled trial of exercise and/or nutrition supplementation.

The exclusion criteria were: 1) severe knee or back pain; 2) severely impaired mobility; 3) impaired cognition (Mini-Mental State Examination (MMSE) score <24); 4) missing baseline data; and 5) unstable cardiac conditions such as ventricular dysrhythmias, pulmonary edema, or other musculoskeletal conditions. Based on the inclusion and exclusion criteria, 331 (18.0%) people were defined as frail. An invitation letter detailing the objectives, methods, and use of personal data in the study were mailed to them, where 131 (39.6%) people participated (Fig. 1).

### Randomization

Randomization was performed after the baseline assessment, and any variable that identified personal information was not included in the randomization process. Computer-generated random numbers were assigned to 131 participants, who were then sorted and equally divided into four groups. IBM SPSS statistics 20.0 was used to generate the random numbers from a normal distribution with mean and standard deviation of any specified variable. The groups were randomly assigned to one of the four interventions: exercise and MFGM supplementation (Ex+MFGM; n = 33), exercise+placebo (Ex+Plac; n = 33), MFGM supplementation (n = 32), or placebo (n = 33) groups. All participants agreed to the group allocations. There was no attempt to equalize the size of the groups based on their characteristics or to recruit subjects with specific characteristics. The co-investigators were blind to the randomization procedure and group allocations, and data collection was conducted by separate physical therapy staff members who were also blind to the allocation of treatments. The principal investigator generated the random allocation sequenced, enrolled the participants, and who assigned participants to the interventions.

### Outcome Measures

Data were collected at baseline, after the 3-month intervention, and at a 4-month follow-up after the completion of the intervention, where measures included interview surveys, body composition assessments using dual-energy x-ray absorptiometry (DXA; Hologic QDR 4500A, USA) [11], and physical function tests.

Frailty Status

The primary outcome of this study was frailty status, which was defined as the presence of 3 or more of the 5 frailty criteria. Frailty reversal rate was defined as the percentage of those who were defined as frail by having at least 3 of the 5 criteria at baseline, but decreased to 2 or below at post-intervention or follow-up.

Each individual frailty criterion, i.e. weight loss, weakness, slow walking speed, exhaustion and low physical activity (described in detail in the inclusion criteria section), was also assessed at baseline, post-intervention, and follow-up.

Interview Survey

Each participant was interviewed face-to-face to assess the individual’s history of chronic illness, medications, falls, fear of falling, fractures, pain, long-term care status, hospitalization, TMIG index [12], exercise habits, urinary incontinence, frequency of going out, and self-rated health.

Body Composition Assessment

Measurements of height and weight were used to calculate BMI (kg/m^2^). Muscle mass, bone mineral density, and body fat mass were determined using DXA.

Functional Fitness Test

Grip strength was assessed using a handheld Smedley-type dynamometer in the dominant hand. Isometric knee extension strength was measured twice using a handheld dynamometer (μTasMF-01, ANIMA, Japan), with the participants seated, knees at a 90 degree angle. The sensor of the measuring device was placed against the anterior side of the ankle in the dominant leg (or the leg with no pain), and the participant was asked to try to extend the knee as hard as possible. The better of two scores were recorded and used for analysis. Walking speed across 5 m was measured as the participants walked on a flat 11 m path with markers at the 3 m and 8 m points. A stop watch was used to measure the time taken to walk between the markers, and the faster of two trials was recorded. A stop watch was also used to measure timed up & go (TUG). Time was measured from the moment the participant stood up from the chair, walked around a cone placed 3 m away, and returned to starting position (seated on the chair), and the faster of two trials was recorded. Assistive walking devices were only used upon the participant’s request, or if the investigators observed any risk of falling.

Hematological Parameters

Serum brain-derived neurotrophic factor (BDNF) concentration was measured with a human BDNF Quantikine ELISA kit (R&D Systems Inc., Minneapolis, MN). Serum insulin-like growth factor (IGF)-1 level was measured with a human IGF-I Quantikine ELISA kit (R&D Systems Inc., Minneapolis, MN). Serum IGF-1 binding protein (IGFBP)-3 level was measured with a human IGFBP-3 Quantikine ELISA kit (R&D Systems Inc., Minneapolis, MN). Serum myostatin was measured with a human Myostatin ELISA kit (Immundiagnostik AG, Bensheim, Germany). All measurements were performed in accordance with the manufacturers’ instructions.

### Intervention

Exercise

The participants in the exercise group were provided with a physical comprehensive training program of moderate intensity. The intensity of the exercises was maintained at approximately 12–14 on the Borg Rate of Perceived Exertion scale. Each exercise class was 60 minutes, held at the TMIG twice per week for 3-months. In order to keep the exercise classes small enough to provide proper instruction, the two exercise intervention groups were further divided into two subgroups, where the participants exercised together within their assigned subgroup in one of four exercise sessions offered per day. There was one instructor for all four classes. Two assistant trainers were present at every class to ensure proper form and observe each participant’s level.

The exercise session included a five minute warm-up, 30 minutes of strengthening exercises, 20 minutes of balance and gait training, followed by a five minute cool-down. The strengthening exercises were performed in a progressive sequence from the seated to standing positions, and progressive resistance was applied through the use of the Thera-bands, and increasing repetition of each time of exercise. Resistance or progression was only increased on a group basis when the participants were able to properly execute each exercise without significant fatigue or loss of proper execution. Each individual’s ability to increase intensity was assessed by the principal investigator, along with the exercise instructor and assistant trainers.

The exercise classes began with seated exercises as the participants were frail older adults, and it provided a secure and stable position. Repetitions of toe raises, heel raises, knee lifts, knee extensions and others, were performed while seated on a chair. To increase difficulty and resistance, participants then performed; hip flexions, lateral leg raises, and repetitions of other exercises while standing upright behind the chair and holding the back of the chair for stability.

Resistance bands were used to further strengthen the upper and lower body. Lower body exercises consisted of leg extensions, hip flexions, and more. Upper body exercises included double-arm pull downs, bicep curls, and others.

Balance and gait training exercises included standing on one leg and multidirectional weight shifts. Participants were instructed on and practiced proper gait mechanics that focused on the maintenance of stability during walking, and increasing stride length, toe elevation of the forward limb, heel elevation of the rear limb, frequency of stepping, and arm swinging.

### MFGM Supplementation

The MFGM group was provided with supplements in pill form, every 2 weeks. MFGM was purchased from Megmilk Snow Brand Co., Ltd. (Sapporo, Japan). The composition of the MFGM was 21.5% protein, 44.0% fat, 26.5% carbohydrate, 33.3% phospholipids (8.29% phosphatidylcholine, 8.56% phosphatidylethanolamine, 2.79% phosphatidylinositol, 3.31% phosphatidylserine, 8.03% sphingomyelin, and others), 6.4% ash, and 1.6% moisture. Each pill contained 167 mg of MFGM, and six pills (total 1 g) were ingested in the mornings, prior to activity. The pills were yogurt-flavored so the participants were able to chew or swallow the pill according to their preference. Participants were asked to fill out a daily diary on which they recorded whether or not they took the full amount of the supplement (if not, how much), and the time of day. These diary sheets were collected every two weeks.

### Placebo

The placebo group followed the same protocol as the MFGM supplementation group; however, the contents of the pill differed. The placebo included whole milk powder instead of MFGM, and the placebo consisted of pills of similar shape, taste, and texture of the MFGM pills. Whole milk powder was purchased from Meiji Milk Products Co. Ltd (Tokyo, Japan). The composition of the milk powder was 26.3% protein, 25.2% fat, 39.5% carbohydrate, 0.286% phospholipids (0.067% phosphatidylcholine, 0.063% phosphatidylethanolamine, 0.037% phosphatidylinositol, 0.033% phosphatidylserine, 0.057% sphingomyelin, and others), 5.7% ash, and 3.3% moisture.

### Data Analysis

Sample size calculations using univariate 1-factor repeated measures ANOVA to examine significant differences in means at baseline, 3 months, and 7 months. Setting the power at 0.80 and an alpha value of 0.05, the total sample size required was estimated to be 112 subjects [13]. When considering a potential attrition rate of 15% [14], 131 subjects were required.

Differences in baseline measures between the groups were measured using a one-way analysis of variance (ANOVA), and chi-square tests were performed on categorical variables. Percent changes in leg muscle mass and gait function were calculated using the formula: % change = ((post-intervention or follow-up value-baseline value)/baseline value)×100), and analysis of covariance (ANCOVA) adjusted for age and baseline frailty score was performed to determine significant differences in percent changes within the groups between baseline to post-intervention and follow-up, with values expressed as differences with 95% confidence intervals. The Scheffe post-hoc method was used when significance was found. The number of frailty criteria was converted to frailty score, with the score corresponding to the number of frailty criteria (eg: 0 for no frailty criteria, 1 for 1 frailty criteria, 2 for 2 frailty criteria, etc). The generalized estimating equation was used to compare the effects between the groups after 3- intervention and at the 7-month follow-up on the change of frailty score. The Kruskal–Wallis test was used to evaluate the differences of the reversal of frailty status between the intervention groups. A post-hoc analysis was performed using the Mann-Whitney method.

To compare the effects of the four intervention groups on frailty after 3-months of intervention and follow-up, multiple logistic regressions were performed, and groups were compared with odds ratios and 95% confidence intervals. All analyses were performed using SPSS software, Windows version 20.0 (SPSS, Inc., Tokyo, Japan).

## Results

There were 3 dropouts (1 death, 1 fracture, 1 spouse care) at the post-survey, 5 dropouts at the follow-up (1 death, 1 femoral neck fracture, 1 hospitalization, 1 moved, 1 declined motivation). The fractures, hospitalizations and deaths were not caused by intervention.

### Baseline Characteristics and Group×Time Interactions

All baseline characteristics were similar between the groups for demographic, physical function, hematological parameters, and interview variables (Table 1).

**Table 1:** Selected variable characteristics of participants at baseline by study group.

Variables*	Ex+MFGM	Ex+Placebo	MFGM	Placebo	
Variables ^*^	(n = 33)			(n = 33)			(n = 32)			(n = 32)			F value^†^	P value
Age (yr)	81.0	±	2.6	81.1	±	2.8	81.0	±	2.8	80.3	±	3.3	0.530	0.662
Height (cm)	147.7	±	5.4	147.8	±	6.7	146.1	±	5.5	144.3	±	5.8	2.625	0.053
Body weight (kg)	46.1	±	7.5	48.6	±	9.0	47.1	±	8.7	47.7	±	8.7	0.503	0.681
Skeletal muscle mass (kg)	13.2	±	1.5	13.8	±	1.7	13.4	±	1.7	13.4	±	1.6	0.691	0.559
Leg muscle mass (kg)	10.1	±	1.1	10.5	±	1.3	10.1	±	1.3	10.1	±	1.2	0.789	0.502
Grip strength (kg)	17.1	±	3.9	17.8	±	2.8	17.5	±	2.7	18.7	±	3.2	1.523	0.212
Knee extension strength (N)	178.8	±	55.2	179.1	±	40.9	185.2	±	52.1	184.7	±	50.1	0.140	0.936
Usual walking speed (sec)	4.5	±	0.9	4.6	±	0.9	4.9	±	1.2	4.7	±	1.5	0.925	0.431
Timed up & go (sec)	7.7	±	1.7	8.2	±	2.0	8.8	±	2.8	8.5	±	3.5	1.096	0.354
BDNF (ng/ml)	6.6	±	1.5	6.8	±	1.4	6.9	±	0.9	6.4	±	1.3	1.167	0.325
Beta-2 microglobulin (mg/ml)	2.6	±	1.0	2.5	±	0.8	2.3	±	0.8	2.6	±	1.2	0.633	0.595
Myostatin (ng/ml)	54.9	±	14.8	48.6	±	11.7	51.6	±	14.7	51.5	±	15.5	0.902	0.443
(IGFBP-3/IGF-1)×100	5.4	±	2.3	4.4	±	2.1	3.9	±	1.3	4.5	±	1.7	2.602	0.056
Number of frailty criteria	3.8	±	0.7	3.6	±	0.7	3.7	±	0.7	3.5	±	0.6	1.429	0.237
Weight loss (%)	72.7			60.6			62.5			45.5				0.155
Exhaustion (%)	60.6			84.8			62.5			60.6				0.099
Low physical activity (%)	90.9			75.8			93.8			90.9				0.106
Low muscle strength (%)	69.7			72.7			65.6			63.6				0.861
Slow walking speed (%)	66.7			60.6			68.8			57.6				0.768
Number of frailty criteria (%)													6.956	0.325
Three	33.3			54.4			43.8			51.5				
Four	48.5			30.3			40.6			45.5				
Five	18.2			15.2			15.6			3.0				
Osteoporosis, yes (%)	51.5			51.5			53.1			42.4			0.936	0.817
Knee osteoarthritis, yes (%)	24.2			21.2			34.4			36.4			2.652	0.448

Note: * Data are presented as M (mean) and SD (standard deviation) for continuous variables, and percentage for categorical variables.

BDNF = brain derived neurotrophic factor, Ex = exercise group

MFGM = milk fat globule membrane

† One-way analysis of variance for continuous variables and chi-square test for categorical variables.

The analysis of changes from baseline, post-intervention and follow-up in muscle mass, physical function and hematological parameters are shown in Table 2. Significant group×time interactions were observed for usual walking speed (P = 0.005), TUG (P<0.001), and (IGFBP3/IGF1) ×100 (P = 0.013). The changes in Ex+MFGM group were significantly greater than MFGM or Placebo group.

**Table 2:** Comparison of muscle mass, physical function, and blood component variables among groups after 3-month intervention and follow-up.

Variables*	Group^†^	Baseline			Post intervention			Follow-up			GEE^‡^	Post hoc analysis ^＃^ (P<0.05)
											(G×T) (P-value)	
Appendicular skeletal	Ex+MFGM	13.20	±	1.50	13.51	±	1.61	13.64	±	1.69	*F* = 0.956	
muscle mass (kg)	Ex+Placebo	13.86	±	1.81	14.04	±	1.77	14.31	±	2.08	(0.416)	
	MFGM	13.20	±	1.79	13.27	±	1.63	13.54	±	1.76		
	Placebo	13.44	±	1.74	13.55	±	1.67	13.70	±	1.75		
Leg muscle mass	Ex+MFGM	10.08	±	1.17	10.30	±	1.21	10.41	±	1.36	*F* = 1.863	
(kg)	Ex+Placebo	10.57	±	1.34	10.34	±	2.40	10.93	±	1.68	(0.140)	
	MFGM	9.99	±	1.28	9.23	±	3.01	10.23	±	1.37		
	Placebo	10.18	±	1.33	10.28	±	1.30	10.39	±	1.38		
Grip strength (kg)	Ex+MFGM	17.19	±	3.79	17.83	±	4.05	17.00	±	3.88	*F* = 0.804	
	Ex+Placebo	17.94	±	3.00	18.36	±	3.28	17.75	±	2.90	(0.495)	
	MFGM	17.81	±	2.35	18.37	±	1.92	16.75	±	2.24		
	Placebo	18.92	±	3.38	19.18	±	3.50	18.08	±	2.92		
Knee extension	Ex+MFGM	187.72	±	49.68	191.52	±	54.81	178.72	±	45.92	*F* = 2.663	
strength (Nm)	Ex+Placebo	179.64	±	40.66	188.45	±	47.82	190.32	±	46.20	(0.053)	
	MFGM	188.68	±	56.89	186.42	±	60.47	181.26	±	51.38		
	Placebo	192.05	±	50.09	194.32	±	54.14	199.95	±	52.65		
Usual walking speed	Ex+MFGM	1.15	±	0.16	1.25	±	0.24	1.23	±	0.21	*F* = 4.592	Ex+MFGM>MFGM
(sec)	Ex+Placebo	1.17	±	0.21	1.26	±	0.27	1.21	±	0.22	(0.005)	
	MFGM	1.10	±	0.22	1.08	±	0.23	1.11	±	0.20		
	Placebo	1.18	±	0.24	1.13	±	0.22	1.18	±	0.23		
Timed up & go	Ex+MFGM	9.63	±	2.15	7.98	±	1.44	6.93	±	1.61	*F* = 9.763	Ex+MFGM, Ex+P>
(sec)	Ex+Placebo	9.89	±	2.27	7.87	±	1.83	7.04	±	1.45	(<0.001)	MFGM, P
	MFGM	10.77	±	2.58	10.53	±	2.77	7.76	±	1.52		
	Placebo	10.44	±	3.79	10.00	±	4.32	7.99	±	3.79		
BDNF	Ex+MFGM	6.60	±	1.54	7.18	±	1.09	7.68	±	1.17	*F* = 1.041	
(ng/ml)	Ex+Placebo	6.37	±	1.44	7.07	±	1.01	7.03	±	1.66	(0.379)	
	MFGM	6.97	±	0.94	7.11	±	1.05	7.39	±	1.47		
	Placebo	6.10	±	1.47	6.36	±	1.31	6.52	±	1.33		
Beta 2 microglobulin	Ex+MFGM	2.67	±	1.14	2.28	±	0.88	2.56	±	0.52	*F* = 0.813	
(mg/L)	Ex+Placebo	2.60	±	0.83	2.53	±	1.32	2.73	±	0.39	(0.490)	
	MFGM	2.18	±	0.65	1.85	±	0.48	2.46	±	0.57		
	Placebo	2.20	±	0.50	2.09	±	0.59	2.49	±	0.39		
Myostatin	Ex+MFGM	54.48	±	14.92	45.75	±	16.02	46.39	±	10.07	*F* = 2.170	
(ng/ml)	Ex+Placebo	49.39	±	11.63	46.48	±	18.11	49.29	±	12.57	(0.097)	
	MFGM	50.42	±	14.82	46.84	±	16.77	48.52	±	12.08		
	Placebo	51.13	±	16.02	48.64	±	17.55	49.59	±	13.75		
(IGFBP3/IGF1)×100	Ex+MFGM	5.50	±	2.28	5.02	±	1.96	4.63	±	1.89	*F* = 3.835	Ex+MFGM>P
	Ex+Placebo	4.18	±	1.46	4.90	±	2.46	5.36	±	1.73	(0.013)	
	MFGM	3.97	±	1.36	4.11	±	1.62	4.24	±	1.51		
	Placebo	4.65	±	1.72	5.38	±	1.93	5.20	±	1.91		
Growth hormone	Ex+MFGM	0.68	±	0.56	1.13	±	0.71	1.07	±	0.46	*F* = 0.301	
(ng/ml)	Ex+Placebo	0.52	±	0.48	0.94	±	0.87	0.97	±	0.59	(0.825)	
	MFGM	0.83	±	0.74	1.24	±	0.90	1.18	±	0.60		
	Placebo	0.72	±	0.64	1.04	±	0.88	1.09	±	0.70		

Note: * Data are presented as mean and standard deviation

† Ex = exercise group

MFGM = milk fat globule membrane

BDNF = brain-derived neurotrophic factor

IGF = insulin-like growth factor

IGFBP = insulin-like growth factor binding protein

P = placebo.

‡ GEE = generalized estimating equation, G = group, T = time.

♯ A post hoc analysis was performed using the Scheffe method (P<0.05).

### Frailty Criteria, Physical Function and Hematological Parameters

The frailty components revealed that weight loss was only reversed between baseline and follow-up in the Ex+MFGM and Ex+Plac groups (Table 3). Reversal of exhaustion was seen across all the groups. Low physical activity was also reversed in all groups, although only the Ex+MFGM group was able to maintain the reversal at follow-up. Slow walking speed was reversed by 42.4% in the Ex+MFGM group between baseline and follow-up, which was significantly greater than the other groups. Change in low muscle strength was not significant.

**Table 3:** Effects of the intervention on each frailty criteria between baseline, post-intervention and follow-up.

Frailty criteria ^†^	Group									
	Ex+MFGM		Ex+Placebo		MFGM		Placebo		P-value ^‡^	Post hoc analysis ^#^
	(n = 33)		(n = 33)		(n = 32)		(n = 32)			(P<0.05)
Weight loss										
Reversal rate from baseline to post-intervention	0.0		-12.1		-18.7		-30.3	*	0.007	Ex+MFGM<MFGM, P
Reversal rate from baseline to follow-up	39.4	*	33.3	*	15.6		-6.1		0.005	Ex+MFGM>MFGM, P; Ex+P>P
Exhaustion										
Reversal rate from baseline to post-intervention	30.3	*	69.7	*	18.7		30.3	*	<0.001	Ex+P>Ex+MFGM, MFGM, P
Reversal rate from baseline to follow-up	33.3	*	42.4	*	25.0	*	-6.1		0.007	Ex+MFGM, Ex+P, MFGM>P
Low physical activity										
Reversal rate from baseline to post-intervention	54.5	*	57.6	*	40.6	*	30.3	*	0.096	
Reversal rate from baseline to follow-up	36.4	*	9.1		9.4		9.1		0.004	Ex+MFGM>Ex+P, MFGM, P
Low muscle strength										
Reversal rate from baseline to post-intervention	6.1		3.0		-12.5		6.1		0.495	
Reversal rate from baseline to follow-up	3.0		-3.1		-9.4		-9.1		0.536	
Slow walking speed										
Reversal rate from baseline to post-intervention	18.2		9.1		-12.5		-3.0		0.247	
Reversal rate from baseline to follow-up	42.4	*	18.2		15.6		0.0		<0.001	Ex+MFGM>Ex+P, MFGM>P

† All data of change (baseline to post-intervention; baseline to follow-up) presented as individual mean percent change.

Reversal rate signifies reversal of each frailty criteria, i.e presented with the criteria at baseline but not at post intervention or follow-up.

* McNemar test P<0.05: within-group percent change between baseline and post-intervention, and baseline and follow-up.

‡ P-values were calculated using Kruskal Wallis for continuous variables.

# Post-hoc analysis was assessed using Mann-Whitney test for continuous variables (P<0.05).

Ex = exercise

MFGM = milk fat globule membrane

P = placebo.

Physical function analysis revealed that walking speed increased in the Ex+MFGM group (14.7%) after the 3-month intervention, which was significantly greater than the MFGM and placebo groups (*P* = 0.026) (Table 4). TUG significantly improved from baseline to post-intervention in both the exercise groups in comparison with the MFGM and placebo groups (P<0.001). Leg muscle mass also increased within the Ex+MFGM and Ex+Plac groups, although significant differences were not seen between the groups.

**Table 4:** Effects of the intervention on each muscle mass and physical function between baseline, post-intervention and follow-up.

Variable	Group													
	Ex+MFGM			Ex+Placebo			MFGM			Placebo			P-value ^‡^	Post hoc analysis ^#^
	(n = 33)			(n = 33)			(n = 32)			(n = 32)				(P<0.05)
Leg muscle mass														
Change from baseline to post-intervention^†^	2.4	±	0.5	1.5	±	0.5	1.2	±	2.5	1.1	±	3.0	0.565	
(95% CI for difference)	(1.3 to 3.5)			(0.3 to 2.3)			(0.2 to 2.2)			(-0.1 to 2.2)				
Change from baseline to follow-up^†^	3.2	±	1.1	3.2	±	1.0	2.5	±	1.1	2.1	±	1.0	0.834	
(95% CI for difference)	(1.1 to 5.4)			(1.2 to 5.3)			(0.29 to 4.7)			(-0.04 to 4.3)				
Grip strength														
Change from baseline to post-intervention^†^	3.9	±	1.6	2.8	±	1.6	3.7	±	2.2	1.4	±	1.2	0.701	
(95% CI for difference)	(0.5 to 7.2)			(-0.5 to 6.0)			(-0.9 to 8.3)			(-1.1 to 3.8)				
Change from baseline to follow-up^†^	-0.2	±	2.6	-0.4	±	1.8	-5.4	±	1.9	-3.4	±	2.5	0.330	
(95% CI for difference)	(-5.6 to 5.1)			(-4.2 to 3.3)			(-9.3 to -1.5)			(-8.6 to 1.8)				
Usual walking speed														
Change from baseline to post-intervention^†^	14.7	±	4.1	9.6	±	3.4	2.1	±	1.9	3.6	±	2.7	0.026	Ex+MFGM>MFGM, P
(95% CI for difference)	(6.4 to 23.1)			(2.7 to 16.4)			(-1.8 to 5.9)			(-1.9 to 9.1)				
Change from baseline to follow-up^†^	14.8	±	3.2	5.3	±	2.5	7.1	±	2.9	6.7	±	2.4	0.070	
(95% CI for difference)	(8.2 to 21.4)			(0.2 to 10.5)			(1.1 to 13.1)			(1.8 to 11.5)				
Timed up & go														
Change from baseline to post-intervention^†^	-14.1	±	2.0	-18.5	±	2.1	-6.1	±	2.6	-3.0	±	2.6	<0.001	Ex+MFGM, Ex+P>MFGM, P
(95% CI for difference)	(-13.8 to -9.9)			(-22.9 to -14.0)			(-11.6 to -0.7)			(-8.3 to 2.3)				
Change from baseline to follow-up^†^	-6.5	±	2.1	-10.2	±	2.5	-4.6	±	3.5	-4.9	±	2.2	0.394	
(95% CI for difference)	(-10.8 to -2.3)			(-15.3 to -5.0)			(-11.8 to 2.5)			(-9.3 to -0.4)				

†All data of change (baseline to post-intervention

baseline to follow-up) presented as mean percent change ± standard error, with 95% confidence intervals (CI)

MFGM = milk fat globule membrane

Ex = exercise.

All mean changes calculated by analysis of covariance adjusted for baseline age and frailty score.

‡ P-values were calculated using ANCOVA for continuous variables.

# Post-hoc analysis was assessed using the Scheffe method for continuous variables (P<0.05).

As seen in Table 5, significant within-group increases in BDNF were observed from baseline to follow-up in both exercise groups. Similarly, significant within-group increases in myostatin were also seen in the Ex+MFGM group between baseline to post-intervention, and follow-up. Decreases in (IGFBP-3/IGF-1) ×100 percent change was only seen in the Ex+MFGM group, while steady increases were seen in the other groups.

**Table 5:** Effects of the intervention on blood components between baseline, post-intervention and follow-up.

Variable	Group													
	Ex+MFGM			Ex+Placebo			MFGM			Placebo			P-value ^‡^	Post hoc analysis ^#^
	(n = 33)			(n = 33)			(n = 32)			(n = 32)				(P<0.05)
BDNF														
Change from baseline to post-intervention^†^	12.1	±	5.5	13.7	±	4.5	2.2	±	2.1	5.0	±	2.9	0.141	
(95% CI for difference)	(0.9 to 23.3)			(4.4 to 23.1)			(-2.1 to 6.5)			(-1.0 to 11.0)				
Change from baseline to follow-up^†^	23.9	±	7.7	17.1	±	10.0	6.9	±	4.5	14.8	±	9.4	0.542	
(95% CI for difference)	(8.1 to 39.7)			(3.6 to 37.9)			(-2.4 to 16.2)			(-4.6 to 34.2)				
Myostatin														
Change from baseline to post-intervention^†^	-17.4	±	2.7	-6.9	±	4.3	-7.9	±	3.7	-3.0	±	5.0	0.083	
(95% CI for difference)	(-23.0 to -11.8)			(-15.9 to 2.2)			(-15.7 to -0.2)			(-13.5 to 7.5)				
Change from baseline to follow-up^†^	-11.1	±	4.2	1.6	±	4.5	-2.2	±	5.6	3.0	±	6.6	0.211	
(95% CI for difference)	(-19.8 to -2.4)			(-7.8 to 10.9)			(-13.9 to 9.5)			(-10.7 to 16.7)				
IGFBP-3/IGF-1														
Change from baseline to post-intervention^†^	-5.3	±	3.7	20.9	±	10.1	8.3	±	7.9	20.7	±	7.8	0.051	
(95% CI for difference)	(-13.0 to 2.3)			(0.2 to 41.6)			(-8.1 to 24.7)			(4.6 to 36.9)				
Change from baseline to follow-up^†^	-7.7	±	8.3	40.1	±	14.2	18.8	±	14.1	22.7	±	10.8	0.036	Ex+MFGM<Ex+P
(95% CI for difference)	(-24.8 to 9.4)			(10.6 to 69.6)			(-10.7 to 48.3)			(0.3 to 45.1)				

† All data of change are presented as mean percent change ± standard error, with 95% confidence intervals (CI)

All mean changes calculated by analysis of covariance (ANCOVA) are adjusted for baseline age and frailty score.

MFGM = milk fat globule membrane

P = placebo; BDNF = brain-derived neurotrophic factor; IGF-1 = insulin-like growth factor 1

IGFBP-3 = insulin-like growth factor binding protein 3.

‡ P-values were calculated using ANCOVA for continuous variables.

# Post-hoc analysis was assessed using the Scheffe method for continuous variables (P<0.05).

### Number of Frailty Criteria and Frailty Status

**Fig 2:**
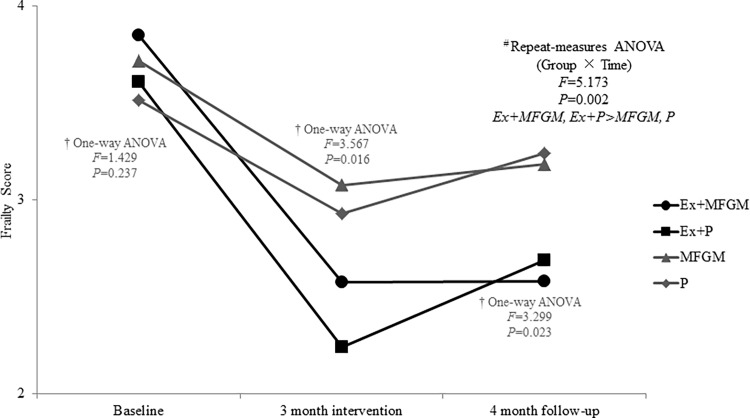
Changes of frailty score between baseline, post-intervention and follow-up. ANOVA = analysis of variance.

The mean number of frailty criteria (out of 5) significantly decreased in all four intervention groups after the 3-month intervention. However, at the 7-month follow-up, the reduction in number of frailty criteria was only able to be maintained in the two exercise groups (Fig. 2). Notably, although the baseline to follow-up change in number of frailty criteria was significant in the Ex+Plac group, the figure clearly depicts an increase in number of frailty criteria from post-intervention to follow-up (as the slope increased). The Ex+MFGM group on the other hand, maintained the same number of frailty criteria at follow-up as post-intervention.

**Fig 3:**
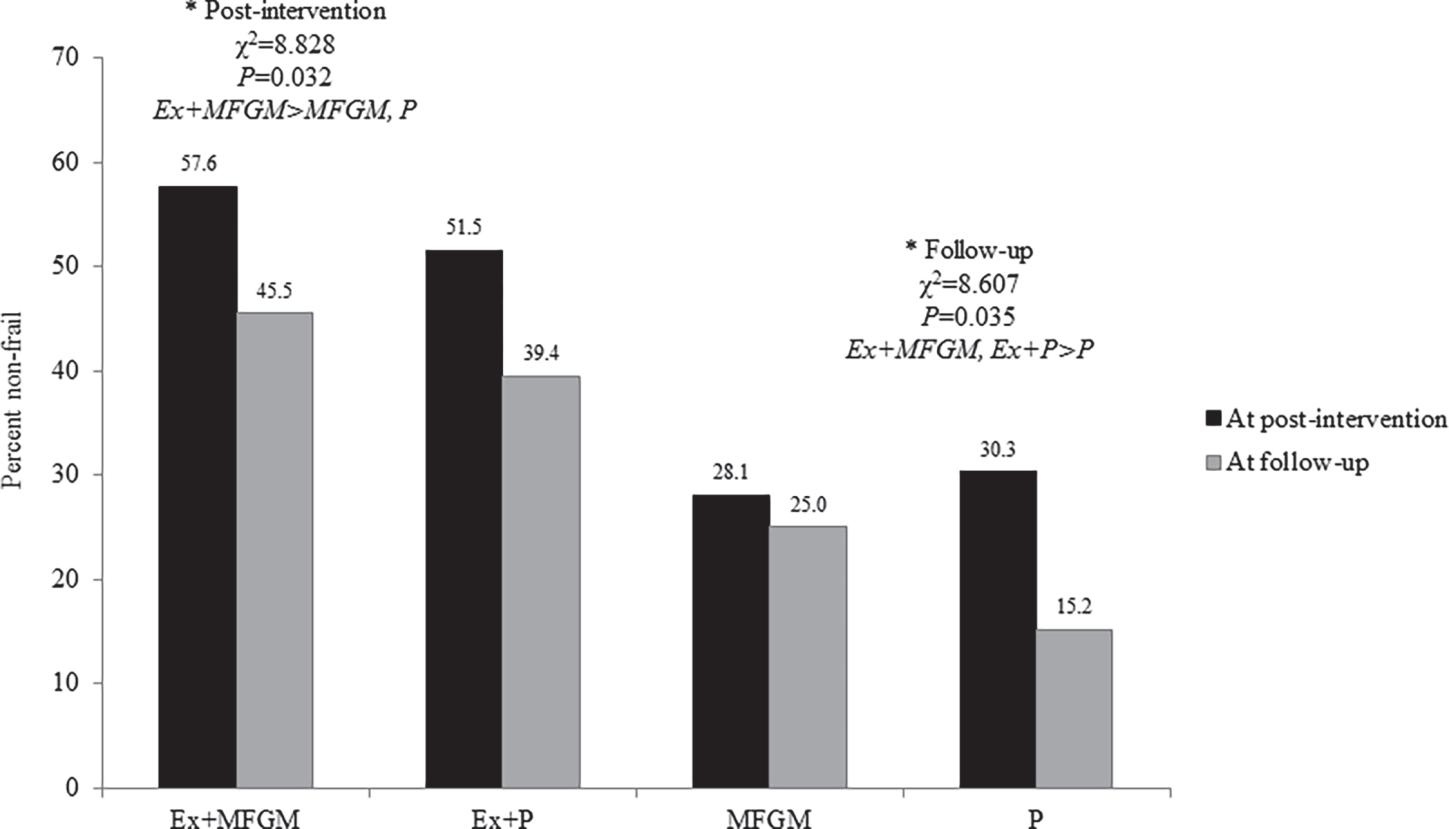
Reversal rates of frailty. Black bar signifies baseline to post-intervention and gray bar signifies baseline and follow-up. Ex = exercise; MFGM = milk fat globule membrane; P = placebo.

Fig. 3 shows that the percentage of non-frail people was significantly higher in the Ex+MFGM (57.6%) than in the MFGM (28.1%) or placebo (30.3%) groups at post-intervention (χ^2^ = 8.827, P = 0.032), and at the follow-up was also significantly greater in the Ex+MFGM (45.5%) and Ex+Plac (39.4%) groups compared with the placebo (15.2%) group (χ^2^ = 8.607, P = 0.035).

### Intervention Type on Frailty Status Reversal

The multiple logistic regression analysis (Table 6) revealed that the Ex+MFGM group had a significant effect on frailty (OR = 3.12, 95%CI = 1.13–8.60) after the 3-month intervention and follow-up improvement of frailty were observed in Ex+Plac (OR = 3.64, 95%CI = 1.12–11.85) and Ex+MFGM (OR = 4.67, 95%CI = 1.45–15.08).

**Table 6:** Adjusted Odds Ratios with 95% Confidence Intervals for frailty at post-intervention and follow-up by intention to treat analysis.

	Adjusted Odds Ratio (95% Confidence Interval)				
Dependent Variable*	MFGM		Exercise+Placebo		Exercise + MFGM
Frailty reversal at post-intervention	0.90 (0.31–2.62)		2.44 (0.89–6.70)		3.12 (1.13–8.60)
Frailty reversal at follow-up	1.87 (0.54–6.47)		3.64 (1.12–11.85)		4.67 (1.45–15.08)

Reference: placebo group

* 0 = frailty, 1 = no frailty

MFGM = milk fat globule membrane.

## Discussion

### Main Findings

The results of this study showed that the 3-month exercise and nutrition supplementation program had an effect on frailty status improvement after the intervention and follow-up. In particular, the frailty components revealed that exhaustion, low physical activity, and slow walking speed were reversed, but low muscle strength did not significantly change. The percentage of non-frail people was significantly higher in the Ex+MFGM (57.6%) than in the MFGM (28.1%) or placebo (30.3%) groups at post-intervention (χ^2^ = 8.827, P = 0.032). Similarly at the follow-up, the percentage of non-frail people was significantly greater in the Ex+MFGM (45.5%) and Ex+Plac (39.4%) groups compared with the placebo (15.2%) group (χ^2^ = 8.607, P = 0.035). The Ex+MFGM group was over 4 times more likely to reverse frailty than the placebo group, and the Ex+Plac group also had a high likelihood of reversing frailty with a significant OR of 3.64 in reference to the placebo group.

### Implications for Frailty Reversal by Exercise and Nutrition Supplementation

Many trials have focused on resistance exercise or nutrition supplementation as treatments to reverse frailty, but the results of these previous trials have not always been consistent. Cameron et al, reported that a multifactorial interdisciplinary intervention can successfully treated frailty status [15]. Furthermore, these results were confirmed, that a 3-month exercise and nutrition intervention resulted in short-term frailty status improvement and long-term effect on bone mineral density and serum vitamin D among Taiwanese community-dwelling elders [14]. Another previous trial showed that although no significant improvements were observed, the intervention group tended to have a better outcome in improving frailty [16]. Poor compliance with the intervention program seemed to be the main reasons for unfavorable outcomes. In the current study on frail elderly women, the Ex+MFGM and exercise groups significantly reduced the number of participants who classified as frail. However the combination of Ex+MFGM may be more beneficial as this group was able to reverse weight loss, low physical activity, and slow walking speed in comparison to all the other groups. The post-intervention OR for frailty reversal was 3.12 in the Ex+MFGM group, which was greater than the 2.44 OR in the Ex+Plac group and 0.90 in the MFGM group. Both the Ex+MFGM (OR = 4.67) and the Ex+Plac (OR = 3.64) groups had significant ORs for the reversal of frailty at follow-up, with the Ex+MFGM group having a greater OR. The OR for MFGM however, was not significant (OR = 1.87). The adjusted ORs for frailty reversal at both post-intervention and follow-up were greater in the Ex+MFGM group than the Ex+Plac and MFGM only groups.

While some studies have focused on frailty status improvement, others placed importance on improving mobility and function. One previous pilot study reported that a home-based older people’s exercise program showed evidence that exercise can reduce the deterioration of mobility in frail older people [17]. Another study demonstrated that resistance training improved muscle strength and size in frail elderly people [18]. These changes were accompanied by improvement in mobility and an increased level of spontaneous physical activity. Multinutrient supplementation did not have any independent or additive effects on the outcomes. Results from clinical trials suggest the use of nutritional consultation as a component in frailty interventions, however not as a stand-alone intervention. One previous study showed that diet and exercise was more effective than diet or exercise alone in improving frailty indicators among 93 obese and frail older adults [19].

Some components of the frailty definition are self-reported while others, like walking speed, are performance based. Performance-based measure should reduce observer bias. Many previous studies have indicated that mobility is a major item in many frailty definitions and appears to predict incident frailty [20]. Mobility is significantly associated with transitions in frailty status [21], and may even be considered as a single-item frailty screening tool [22]. In this study, we analyzed walking ability in frail women in more detail.

Subgroup analysis of the frailty components showed that usual walking speed increased in the Ex+MFGM group by 0.25±0.07 m/s (mean percent change 27.0%, 95% CI for difference = 9.69 to 44.26) at post intervention and 0.22±0.05 m/s (mean percent change 23.9%, 95% CI for difference = 13.04 to 34.82) at follow-up, and in the Ex+Plac group by 0.21±0.05 m/s (mean percent change 22.5%, 95% CI for difference = 9.89 to 35.19) after the three-month intervention and 0.09±0.06 m/s (mean percent change 11.0%, 95% CI for difference = -5.6 to 27.7) after the follow-up. However, the changes were not significant in the MFGM and Placebo groups. The improvements in walking speed observed in both exercise groups are clinically significant, as the Society on Sarcopenia, Cachexia, and Wasting Disease stated that an improvement in gait speed of at least 0.1 m/s can be considered as such [23]. Exercise alone or combined exercise and MFGM supplementation was effective for improving walking ability in frail women with walking speed less than 1.0 m/s. Further analysis showed that the change in walking speed among participants with speeds less than 1.0 m/s, based on the frailty criteria, was 0.13 m/s at post-intervention and 0.12 m/s at follow-up, and in those with walking speeds greater than 1.0 m/s was 0.05m/s at post-intervention and 0.06 m/s at follow-up. Greater improvements were observed in participants with slow walking speed, and smaller improvements were seen in those with faster walking speeds. The improvements in physical activity and exhaustion seen may have possibly been related to the improvements in walking ability.

Researchers have suggested that grip strength testing is likely to be increasingly used in clinical setting, for example in the assessment of sarcopenia, frailty and undernutrition in hospitalized older people [24,25]. In the current study, statistically significant changes in grip strength were not observed in the intervention groups. This may be due to the content of the exercise program. Grip strength is a measure of upper body strength; however the focus of the exercise program was placed on the lower body. Therefore since grip strength as an outcome variable may not have been a particularly suitable measure of strength of the exercise intervention conducted, lower extremity strength measures should also be used in future studies.

Unintentional weight loss is one criterion included in the definition of frailty based on Fried’s phenotype; however, its use may not be appropriate in evaluating the effects of interventions on frailty status. During the three month intervention in this study, the change in weight was -3.0∼2.0 kg, and -3.1∼3.5 kg during the follow-up. Whether these changes were unintentional or due to the exercise are unclear and further research is necessary.

Although the Fried frailty phenotype has been validated and modified for use in several published reports, cognitive and psychological factors, which have known association with functional decline and disability, were not included in the frailty phenotype [26]. Several clinical studies show that blood brain-derived neurotrophic factor (BDNF) levels are reduced in Alzheimer’s disease [27], mild cognitive impairment [26], and major depressive disorder [28]. In the current study, changes in BDNF levels in the Ex+MFGM and Ex+Plac groups between baseline and follow-up were 23.9% and 17.1%, respectively. These results were significant within each group, hence exercise increased BDNF levels, which is in agreement with previous findings [29,30]. Although depression and cognitive function were not investigated, exercise alone or in congruence with nutrition intervention may potentially be beneficial for the improvement of depression and cognitive function. Further research is necessary.

Myostatin is a significant negative regulator of skeletal muscle development and size [31]. Some studies showed decreases in myostatin protein levels in postmenopausal women and middle-aged men after aerobic exercise [32,33]. However, the effects of modest exercise and nutrition supplementation on myostatin in skeletal muscle remain largely unaddressed in elderly adults. The within group decreases in myostatin from baseline to post-intervention, and follow-up in the Ex+MFGM group was 17.4%, and 11.1%, respectively. Although the decreases obtained in this study were less than those reported by Ryan et al [33], who conducted a 6 month, 3 days per week intervention, the Ex+MFGM intervention significantly decreased myostatin in community-dwelling elderly women.

The insulin-like growth factor (IGF-1) pathway is thought to play major roles in exercise induced muscle hypertrophy and maintenance of muscle, and several groups have explored the effects of resistance training on IGF-1 in older persons, although the results have been inconclusive [34–36]. Several studies have shown that resistance training does not alter IGF-1 or IGFBP-3 in elderly subjects [37,38]. In contrast, Parkhouse et al reported that resistance training increased IGF-1 in older women who had low bone mineral density, and the IGFBP-3/IGF-1 ratios significantly decreased from resistance training [39]. The authors suggested that potentially more IGF-1 were bound to IGFBP-3, possibly contributing to the significant strength gains observed with resistance training in the elderly population. In the current study, IGFBP-3/IGF-1 ratios only decreased in the Ex+MFGM group, while all the other groups showed increases.

The effects of MFGM alone on frailty reversal, physical function, and biomarkers were minimal in this study. However, MFGM alone did show reversal in exhaustion and low physical activity, although this may be attributed to patient-provider interaction where simply participating in this trial motivated participants in both the MFGM and placebo groups to live healthier lifestyles by increasing their daily physical activity. However, Haramizu et al suggested that the phospholipid and sphingolipids in MFGM together with exercise can improve muscle function deficits through neuromuscular development, as well as neuromuscular junction formation [10]. The authors suggest that the increase in plasma adiponectin observed could have contributed to the greater muscle force and whole-body energy expenditure with exercise and MFGM supplementation in mice. Furthermore, the combination of exercise and MFGM increased the levels of MRNA expression for MyoD and myogenin, which may help to improve NMJ formation, hence improving contractile function of the muscles. This data shows that leg muscle mass and walking speed increase after the intervention in the combine group (Tables 4 and 5), and myostatin levels improved with the intake of MFGM. However, statistically significant additive effects of MFGM with exercise were not observed.

## Strengths and Limitations

Strength of this study is in the randomized controlled trial design, and also that it is a double blind and placebo-controlled trial. This study is also the first to explore the effects of an exercise and nutrition intervention on frailty status as well three different aspects: body composition, functional fitness, and hematological parameters.

This study has several limitations. This study focused on elderly women; hence the results of this study cannot be generalized for elderly men. Typically, based on Fried’s criteria for frailty, walking speed and grip strength are stratified by height and BMI, respectively. The height cut point used by Fried was 159 cm for women, however only 29 (1.6%) out of 1,835 women were taller than 159 cm, in this Japanese population. Similarly, only 57 (3.1%) people out of the 1,835 had a BMI of greater than 29 kg/m^2^, which was the highest quartile cut point used by Fried. Therefore, the cut points used for an American population may be difficult for use in a Japanese population. Further research and analysis of frailty with these cut points in a larger sample of Japanese people is necessary. Future research should also look to follow the participants for a longer follow-up period. Furthermore, readers should be aware of the placebo effects and bias as only the nutritional supplementation could be blinded and exercise could not.

Finally, while statistically significant additive effects of MFGM with exercise could not be confirmed in this population, this data suggested that the nutritional supplementation may be beneficial for the improvement of frailty in elderly women. Generalization of these findings requires the consideration that only 39.6% of all the potential participants who were defined as frail participated in the intervention. The 60.4% of non-participants (or those excluded) had greater mobility impairments. The results of this study should be interpreted with consideration of such selection bias. Further investigation in larger samples is necessary.

## Conclusion

This study found that Ex+MFGM showed greater odds of frailty reversal, suggesting that this nutritional supplementation may perhaps be beneficial for the improvement of frailty in elderly women. However, statistically significant additive effects of MFGM with exercise could not be confirmed in this population. Further analysis showed that Ex+MFGM significantly reversed four of the five components in Fried’s frailty phenotype, where improvements in walking ability due to the exercise may have played a major role. The improvements in muscle mass and hematological parameters may have been mediating factors in the improvement in walking ability, possibly leading to the reversal of frailty status, however further research on a larger sample is necessary.

## Supporting Information

S1 CONSORT Checklist. (DOC)

S1 Protocol. (DOCX)
