# Supplementary material for: Effects of Exercise and Milk Fat Globule Membrane (MFGM) Supplementation on Body Composition, Physical Function, and Hematological Parameters in Community-Dwelling Frail Japanese Women: A Randomized Double Blind, Placebo-Controlled, Follow-Up Trial
Source: PLoS One. 2015 Feb 6;10(2):e0116256. doi: 10.1371/journal.pone.0116256 (PMC4319727; doi:10.1371/journal.pone.0116256)
Supplement: S1 Protocol. — (DOCX) [file pone.0116256.s002.docx]

**Project Title**: The effects of a comprehensive intervention on the improvement of physical function in community-dwelling frail elderly people.

Principal Investigator:

Hunkyung Kim, PhD

**Scientific Protocol**

**Summary**

Distribution of Roles: H Kim principal investigator; H Yoshida medical check; M Kim physical function measurement, exercise instruction and management.

Objective: To investigate the effects of a comprehensive intervention program on physical function improvement in community-dwelling older people.

Subjects: Participants will be recruited from two cohorts of ongoing longitudinal health surveys. Among those who completed the health survey in November 2009 (Cohort 1) and October 2010 (Cohort 2), we will invite the older adults who are classified as being frail according to Fried’s frailty criteria [[1](#_ENREF_1)], to participate in this intervention study.

Method: An information session will be held (August 1, 2011), where the protocol will be explained to the participants, and written informed consent will be obtained. After consent is received, the participants will be randomly placed in the following four groups for a 12 week intervention.

- Group 1 (approx. 35 people) – exercise and nutrition 1
- Group 2 (approx. 35 people) – exercise and nutrition 2
- Group 3 (approx. 35 people) – nutrition 1
- Group 4 (approx. 35 people) – nutrition 2

The exercise intervention will be held twice a week, for 60 minutes per session, where the comprehensive program will focus on the improvement of physical function. The nutrition 1 intervention will include taking 6 tables of milk fat globule membrane (MFGM) every day for 12 weeks, and nutrition 2 will follow the same procedure but with placebo tablets. The health education group will receive a lecture once a month. We will assess the participants and compare each group on strength (muscle strength, walking speed, etc), quality of life, blood components, and physical activity before and after the intervention.

**Introduction/Rationale**

Frailty has been an area of focus in research since the early 2000s. The study of frailty may give health care professionals insight into the loss of independence in the elderly people after illness and hospitalization. Since frailty is not present in all elderly people, this suggests that the condition can be prevented or treated [[2](#_ENREF_2)]. There have been various definitions of frailty, but perhaps the most commonly used definition is based on Fried’s phenotype [[1](#_ENREF_1)]. Fried defines frailty as the presence of three of the following five criteria: unintentional weight loss, muscle weakness, exhaustion, slow walking speed, and low physical activity. We plan on performing a randomized controlled trial on community-dwelling women based on this definition.

While frailty prevention has been investigated from many different angles and areas, exercise has been a main focus. The effects of exercise on muscle weakness, walking speed, and physical activity are well established in the literature [[3](#_ENREF_3),[4](#_ENREF_4)]. Furthermore, previous studies have reported that exercise is effective in improving physical function in frail older adults [[5](#_ENREF_5),[6](#_ENREF_6)]. However, these studies use different definitions of frailty and there is difficulty in reaching a consensus when these definitions vary. Further study is necessary to investigate the effects of exercise on a commonly used definition of frailty.

Exercise in combination with nutritional supplementation has been reported to be effective in improving physical function in elderly people [[5](#_ENREF_5)]. Milk proteins are high in amino scores and are highly digestible proteins [[7](#_ENREF_7)]. Recent findings have suggested that consumption of whole milk after resistance training can be beneficial for muscle protein synthesis, which can lead to improved muscle mass [[8-10](#_ENREF_8)]. In recent years, the composition and properties of milk fat globule protein (MFGM) has grown significantly. MFGM is a complex structure composed of different protein and lipid components with specific nutritional characteristics. MFGM consists of phospholipids and sphingolipids which are suggested to have various beneficial effects such as cell absorption processes, molecular transport systems, development and growth, memory, stress responses, and myelination in the central nervous system [[11-14](#_ENREF_11)]. Since deterioration of the myelin sheath with aging may lead to the decline in conduction velocity of motor neurons, hence muscle strength and mass, these lipids are very important to study. A review article has also summarized that sphingolipids may inhibit colon carcinogenesis, reduces LDL cholesterol while elevating HDL cholesterol in animal studies [[15](#_ENREF_15)]. There are very few studies that investigate the effects MFGM on humans. This particular nutritional supplementation may have many health benefits; however it is still under-studied.

The combination of exercise and MFGM as an intervention has never been studied. The potential health benefits of each of the preventive measures for frailty have been studied independently, but never together. This unique study will provide insight into the prevention of frailty, a debilitation condition among elderly people.

**Methods**

**Design**

A randomized double blind, placebo-controlled, follow-up trial.

**Subject recruitment**
Participants will be recruited from two cohorts of ongoing longitudinal health surveys. Among those who completed the health survey in November 2009 (Cohort 1) and October 2010 (Cohort 2), we will invite the older adults who are classified as being frail according to Fried’s frailty criteria [[1](#_ENREF_1)], to participate in our study. In November 2009, out of 1,447 women aged 77 and older who were invited to take part in the annual general health survey at the Tokyo Metropolitan Institute of Gerontology, 974 people participated. In October 2010, 1,458 people aged 74 and older were invited to the general health survey, and 861 participated. A total of 1,835 people took part in the health survey in 2009 and 2010, which will be considered the sample population for this study, from which frailty will be assessed. Those considered frail will be sent an invitation to participate in this study by mail. We will ask all those willing to participate to an information session (planned on August 1, 2011), and those who consent to participation and submit written informed consent will be the participants of the study. Participation is solely on volunteer basis and the participants will not receive any kind of monetary or gift compensation. The exercise program aims to improve physical function and the health education classes will provide information sessions on general health.

**Pre-intervention Survey**

1. Body Composition

Body fat, percent body fat, skeletal muscle mass will be determined via dual energy x-ray absorptiometry (Hologic QDR 4500A, USA).

1. Anthropometric Measures

Height will be measured using a satiometer. Participants will remove their shoes and socks and stand upright with their heels against the bottom of the stadiometer. Participants will be instructed to focus on a point straight ahead and pull down their chin if their faces are angled upwards. If the participant has difficulty standing upright, a straight edge object will be used to extrapolate the line from the head of the stadiometer to the crown of the participant’s head. Staff members performing the assessment will also attempt to align the participants’ head to the center if they tilt to the left or right. To measure weight, participants will be asked to step onto a scale without shoes or socks. Measurements of height and weight will be used to calculate BMI (kg/m^2^).

1. Interview Survey

Interviews will be conducted to assess chronic diseases, activities of daily living (ADL), instrumental activities of daily living (IADL), Tokyo Metropolitan Institute of Gerontology (TMIG) Index of Competence, fall history, urinary incontinence, frequency of going out doors, knee pain, QOL.

1. Physical Function Measures

Grip strength will be measured using a handheld Smedly-type dynamometer in the dominant hand. Instructors will adjust the width of the hand grip to make it comfortable for the participants. The participants will stand upright, with feet shoulder width apart, holding the dynamometer in the dominant hand, and the instructor will signal when to begin and when to end. Encouragement to grip harder will be given along with reminders to keep breathing so as to not increase blood pressure. Isometric knee extension strength will be measured twice using a handheld dynamometer (μTasMF-01, ANIMA, Japan), with the participants seated, knees at 90 degree angles. The sensor of the measuring device will be placed against the anterior side of the ankle in the dominant leg or the one without pain. The participants will be asked to try to extend the knee with as much force as possible. The better of two scores will be recorded. One leg standing time with eyes open will be measured to assess balance. Participants will stand one meter away from a white board with a red “X” placed at eye level and be asked to gaze at the mark while standing on one leg. The lifted leg cannot touch the standing leg. Participants may spread their arms out to regain balance. The best of two trials will be recorded. Walking speed will be measured across five meters on a flat 11 meter path with markers at the 3 and 8 meter points. A stop watch will be used to measure the time taken to walk between the markers, and the faster of two trials will be recorded. Timed up & go will also be measured. A stop watch will be used to measure the time from the moment the participants stand from the chair, walk around a cone placed 3 meters away, and return to starting position (seated on the chair). The faster of two trials will be recorded. Assistive walking devices will be allowed upon the participant’s request or if the investigators observe any fall risk.

1. Physical Activity

The Lifecorder EX will be used to evaluate number of steps as well as the intensity and duration of physical activity. The participants will be asked to secure the Lifecorder on the left or right hip, around the anterior superior iliac crest, on their clothing. The Lifecorder will be placed on the hip when the participant wakes up and taken off at night when they go to bed, although the device will be temporarily removed in activities involving water such as swimming or taking a bath.

1. Blood Components

Serum brain-derived neurotrophic factor (BDNF) concentration will be measured with a human BDNF Quantikine ELISA kit (R&D Systems Inc., Minneapolis, MN). Serum insulin-like growth factor (IGF)-1 level will be measured with a human IGF-I Quantikine ELISA kit (R&D Systems Inc., Minneapolis, MN). Serum IGF-1 binding protein (IGFBP)-3 level will be measured with a human IGFBP-3 Quantikine ELISA kit (R&D Systems Inc., Minneapolis, MN). Serum myostatin will be measured with a human Myostatin ELISA kit (Immundiagnostik AG, Bensheim, Germany). All measurements will be performed in accordance with the manufacturers’ instructions.

Intervention

1. Exercise

The participants in the exercise group will be provided with a physical comprehensive training program of moderate intensity. The intensity of the exercises will be maintained at approximately 12-14 on the Borg Rate of Perceived Exertion (RPE) scale. Each exercise class will be 60 minutes, held at the TMIG twice per week for 3-months. In order to keep the exercise classes small enough to provide proper instruction, the two exercise intervention groups will be further divided into two subgroups, where the participants exercise together within their assigned subgroup in one of four exercise sessions offered per day. There will be one instructor for all four classes. Two assistant trainers will be present at every class to ensure proper form and observe each participant’s level.

The exercise session will include a five minute warm-up, 30 minutes of strengthening exercises, 20 minutes of balance and gait training, followed by a five minute cool-down. The participants will aim to perform the strengthening exercises in a progressive sequence from the seated to standing positions, and progressive resistance will be applied through the use of the Thera-bands, and increasing repetition of each time of exercise. Resistance or progression will be only increased on a group basis when the participants are able to properly execute each exercise without significant fatigue or loss of proper execution. Each individual’s ability to increase intensity will be assessed by the principal investigator, along with the exercise instructor and assistant trainers.

*Chair exercise:* The exercise classes will begin with seated exercises as the participants are frail older adults, and it provides a secure and stable position. Repetitions of toe raises, heel raises, knee lifts, knee extensions and others, are planned to be performed while seated on a chair. To increase difficulty and resistance, participants will perform more difficult exercises such as; hip flexions, lateral leg raises, and repetitions of other exercises while standing upright behind the chair and holding the back of the chair for stability.

*Exercises using a resistance band (Thera-Band):* Resistance bands will be used to further strengthen the upper and lower body. Lower body exercises consist of leg extensions, hip flexions, and more. Upper body exercises include double-arm pull downs, bicep curls, and others.

*Balance and gait training:* Exercises include standing on one leg and multidirectional weight shifts. Participants will be instructed on and practice proper gait mechanics focusing on the maintenance of stability during walking, and increasing stride length, toe elevation of the forward limb, heel elevation of the rear limb, frequency of stepping, and arm swinging.

1. MFGM supplementation

The MFGM group will be provided with supplements in pill form, every 2 weeks. MFGM was purchased from Megmilk Snow Brand Co., Ltd. (Sapporo, Japan). The composition of the MFGM is 21.5% protein, 44.0% fat, 26.5% carbohydrate, 33.3% phospholipids (8.29% phosphatidylcholine, 8.56% phosphatidylethanolamine, 2.79% phosphatidylinositol, 3.31% phosphatidylserine, 8.03% sphingomyelin, and others), 6.4% ash, and 1.6% moisture. Each pill contains 1 g of MFGM, and six pills will be ingested in the mornings, prior to activity. The pills are yogurt-flavored so the participants will be able to chew or swallow the pill according to their preference. In order to keep records of the supplement intake, participants will fill out a daily diary on which they will record whether or not they took the full amount of the supplement (if not, how much), and the time of day. These diary sheets will be collected every two weeks.

1. Combined Exercise + MFGM supplementation

Participants will be asked to follow both protocols of the exercise and MFGM interventions in congruence. Every two weeks, the participants will be provided with the supplementation and the daily diaries will be collected when they arrive at their exercise class.

1. Placebo

The placebo group will follow the same protocol as the MFGM supplementation group; however, the contents of the pill will differ. The placebo includes whole milk powder instead of MFGM, and the placebo consists of pills of similar shape, taste, and texture of the MFGM pills. Whole milk powder will be purchased from Meiji Milk Products Co. Ltd (Tokyo, Japan). The composition of the milk powder is 26.3% protein, 25.2% fat, 39.5% carbohydrate, 0.286% phospholipids (0.067% phosphatidylcholine, 0.063% phosphatidylethanolamine, 0.037% phosphatidylinositol, 0.033% phosphatidylserine, 0.057% sphingomyelin, and others), 5.7% ash, and 3.3% moisture.

**Post-intervention Survey**

The same protocol as the pre-intervention survey will be followed post-intervention.

**Follow-up Survey**
The participants will return to the TMIG for a follow-up survey 4 months after the post-intervention survey. No specific instructions will be given to the participants during the follow-up period. The same protocol will be followed for each measure and survey item as the pre- and post-interventions.

**Adverse events**

Adverse events are defined as any untoward medical occurrence associated with the intervention, whether or not considered directly related to the intervention. In the case of minor events, the participants will stop the intervention immediately. In more major cases, the participants will be sent to the emergency room at the hospital immediately if events occur during the exercise classes. All adverse events will be followed and the participants will be supported by medical staff at the TMIG.

**Dropouts**

Participants who could not attend or complete 60% of the exercise or MFGM supplementation will be excluded from the data analysis.

**Data Analysis**

Differences in baseline between the groups will be analyzed using analysis of variance (ANOVA) for continuous variables, and chi-square tests for categorical variables. The generalized estimating equation will be analyzed to compare the effects of the intervention between the groups after the three month intervention and at the follow-up on frailty status. Further analyses using multiple logistic regressions will be used to obtain odds ratios for frailty in each group with the placebo as the reference.

**Ethical Considerations**

**Individual protection (Privacy, risks)**

1. Exercise intervention: Programs similar to the one described above have been done previously. Our experience shows that this is a safe program for elderly people.
2. Nutritional Supplementation Intervention: The MFGM that will be used in this study in a substance found in breast milk or cow milk. The triglyceride core of the fat globules in milk is surrounded by a thin membrane, which is the MFGM. The phospholipids and sphingolipids are located mainly in the MFGM, and these lipids have been shown to have beneficial health effects [[13](#_ENREF_13)]. Recent studies have also shown that the consumption of whole milk after resistance training can improve muscle protein synthesis [[8-10](#_ENREF_8)] (Elliot et al 2006, Josse et al 2010, Wilkinson et al 2007). Hence, the intake of MFGM in combination with exercise may have benefits for muscle strength and mass. This MFGM has been used commercially from companies such as Suntory and Nisshin, and is safe for the promotion of health in frail older adults.

**Consent**

People who are classified as frail based on Fried’s phenotype will be invited to participate in this intervention study. An information session will be held, thoroughly explaining the interventions to the potential participants. All the risks and benefits of each program will be presented. The attendees will be informed that they may stop participation whenever they please, and that it is volunteer basis as no form of compensation will be given. The people who agree to participation will sign and date an informed consent form. The study is conducted in agreement with the Declaration of Helsinki.

**Potential Risks and Benefits**

There is potential risk of minor injuries or pain resulting from performing exercises that elderly people are unaccustomed to doing. The MFGM supplementation contains substances similar to milk, and any participant who has dietary restrictions may have to refrain from taking this supplement. The participant is free to discontinue the study when they choose.

While the effects of the comprehensive intervention program for frail elderly people is still understudied, the exercise and nutrition intervention has a high potential of improving muscle mass, and physical function while also providing a base for further long-term care program development.

**Cross-over Design**

Participants in the first phase of the trial (September 12, 2011~December 1, 2011) will be followed for 4 months post intervention. After the follow-up period, the second phase of the trial will begin (March, 2012~June, 2012), where the exercise group will be crossed over with the nutrition group and vice versa, and the control group crossed over with the exercise+nutrition group and vice versa. There will be no changes to the protocols between the trial phases.

**End of Study**

The study will terminate after Phase 2 of the trial, i.e. after the cross over and subsequent follow-up, 14 months after the first baseline testing in 2011. Any unexpected premature termination of the study will be notified along with the reasons to the TMIG ethics committee. This study will not involve any longer term care of participants beyond the 14 months. Any required care as a consequence of the intervention or survey during the 14 months will be provided for by the investigators at the TMIG hospital.

**Access to Data**

Hard copies of all the data will be locked in cabinets, in a locked storage room. The paper copies will be made into PDF documents and shredded after 5 years of storage. The PDF data will be supervised and managed by the TMIG. The digital data will be kept on a password protected file on a USB flash drive, with a backup copy made onto another USB flash drive managed by the principal investigator, and will only be accessed by the investigators of this study. All participants identifying information will be coded to keep each person’s privacy. The data obtained in this study will be available to the public and published, independent of the outcomes. Data will not be obtained from any government agency.

**Reference**

1. Fried LP, Tangen CM, Walston J, Newman AB, Hirsch C, et al. (2001) Frailty in older adults: evidence for a phenotype. Journals of Gerontology Series A, Biological Sciences and Medical Sciences 56: M146-156.

2. Ahmed N, Mandel R, Fain MJ (2007) Frailty: an emerging geriatric syndrome. American Journal of Medicine 120: 748-753.

3. Liu CJ, Latham NK (2009) Progressive resistance strength training for improving physical function in older adults. Cochrane Database Syst Rev: CD002759.

4. Peterson MD, Rhea MR, Sen A, Gordon PM (2010) Resistance exercise for muscular strength in older adults: a meta-analysis. Ageing Res Rev 9: 226-237.

5. Fiatarone MA, O'Neill EF, Ryan ND, Clements KM, Solares GR, et al. (1994) Exercise training and nutritional supplementation for physical frailty in very elderly people. N Engl J Med 330: 1769-1775.

6. Li CM, Chen CY, Li CY, Wang WD, Wu SC (2010) The effectiveness of a comprehensive geriatric assessment intervention program for frailty in community-dwelling older people: a randomized, controlled trial. Archives of Gerontology and Geriatrics 50 Suppl 1: S39-42.

7. Phillips SM, Tang JE, Moore DR (2009) The role of milk- and soy-based protein in support of muscle protein synthesis and muscle protein accretion in young and elderly persons. Journal of the American College of Nutrition 28: 343-354.

8. Elliot TA, Cree MG, Sanford AP, Wolfe RR, Tipton KD (2006) Milk ingestion stimulates net muscle protein synthesis following resistance exercise. Med Sci Sports Exerc 38: 667-674.

9. Josse AR, Tang JE, Tarnopolsky MA, Phillips SM (2010) Body composition and strength changes in women with milk and resistance exercise. Medicine and Science in Sports and Exercise 42: 1122-1130.

10. Wilkinson SB, Tarnopolsky MA, Macdonald MJ, Macdonald JR, Armstrong D, et al. (2007) Consumption of fluid skim milk promotes greater muscle protein accretion after resistance exercise than does consumption of an isonitrogenous and isoenergetic soy-protein beverage. Am J Clin Nutr 85: 1031-1040.

11. Astaire JC, Ward R, German JB, Jimenez-Flores R (2003) Concentration of polar MFGM lipids from buttermilk by microfiltration and supercritical fluid extraction. Journal of Dairy Science 86: 2297-2307.

12. Murgiano L, Timperio AM, Zolla L, Bongiorni S, Valentini A, et al. (2009) Comparison of milk fat globule membrane (MFGM) proteins of Chianina and Holstein cattle breed milk samples through proteomics methods. Nutrients 1: 302-315.

13. Oshida K, Shimizu T, Takase M, Tamura Y, Yamashiro Y (2003) Effects of dietary sphingomyelin on central nervous system myelination in developing rats. Pediatric Research 53: 589-593.

14. Horrocks LA, Farooqui AA (2004) Docosahexaenoic acid in the diet: its importance in maintenance and restoration of neural membrane function. Prostaglandins Leukotrienes and Essential Fatty Acids 70: 361-372.

15. Vesper H, Schmelz EM, Nikolova-Karakashian MN, Dillehay DL, Lynch DV, et al. (1999) Sphingolipids in food and the emerging importance of sphingolipids to nutrition. Journal of Nutrition 129: 1239-1250.

*Supplementary original documents*

Ethical Committee Approval

*
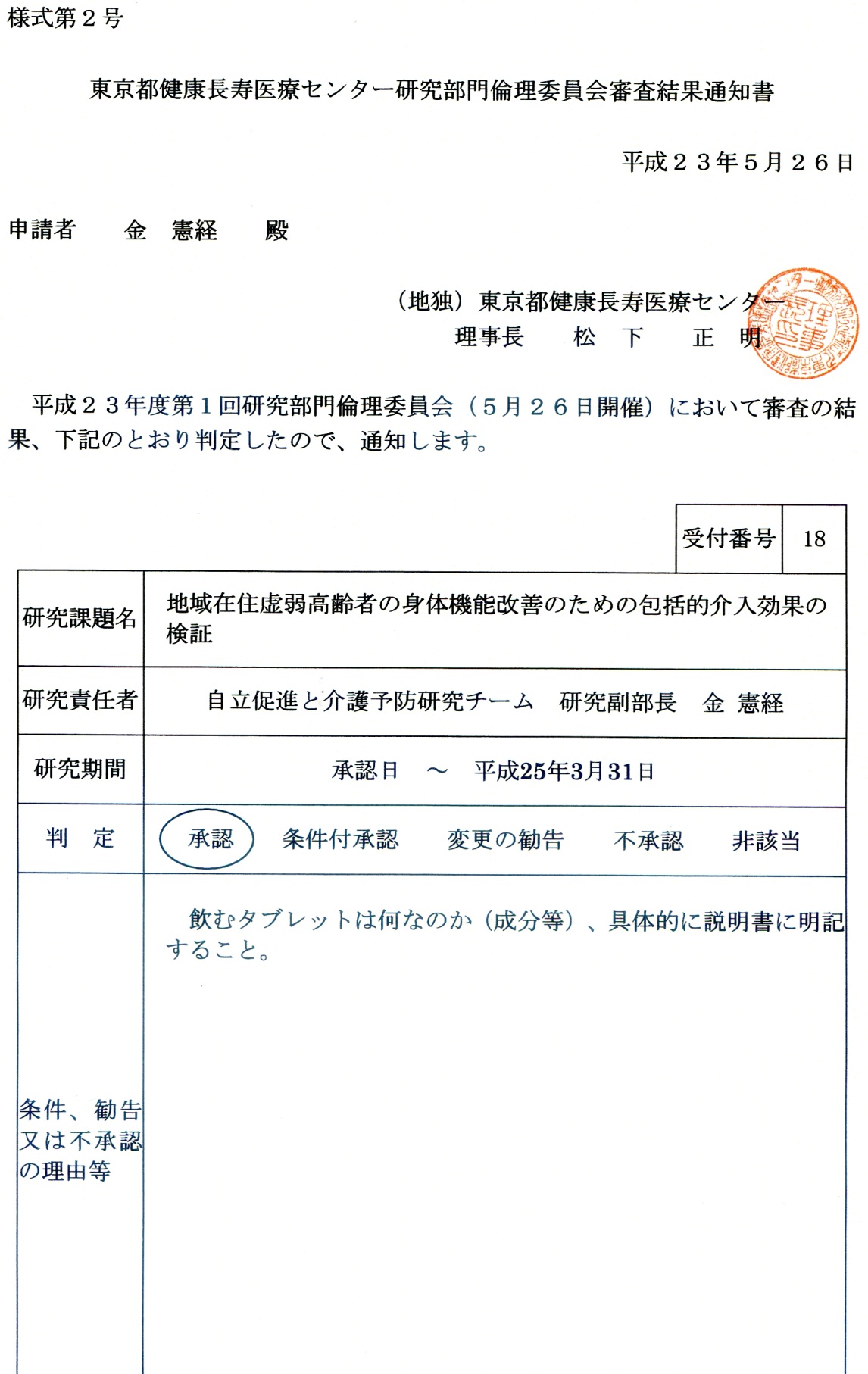
*

Invitation Letter


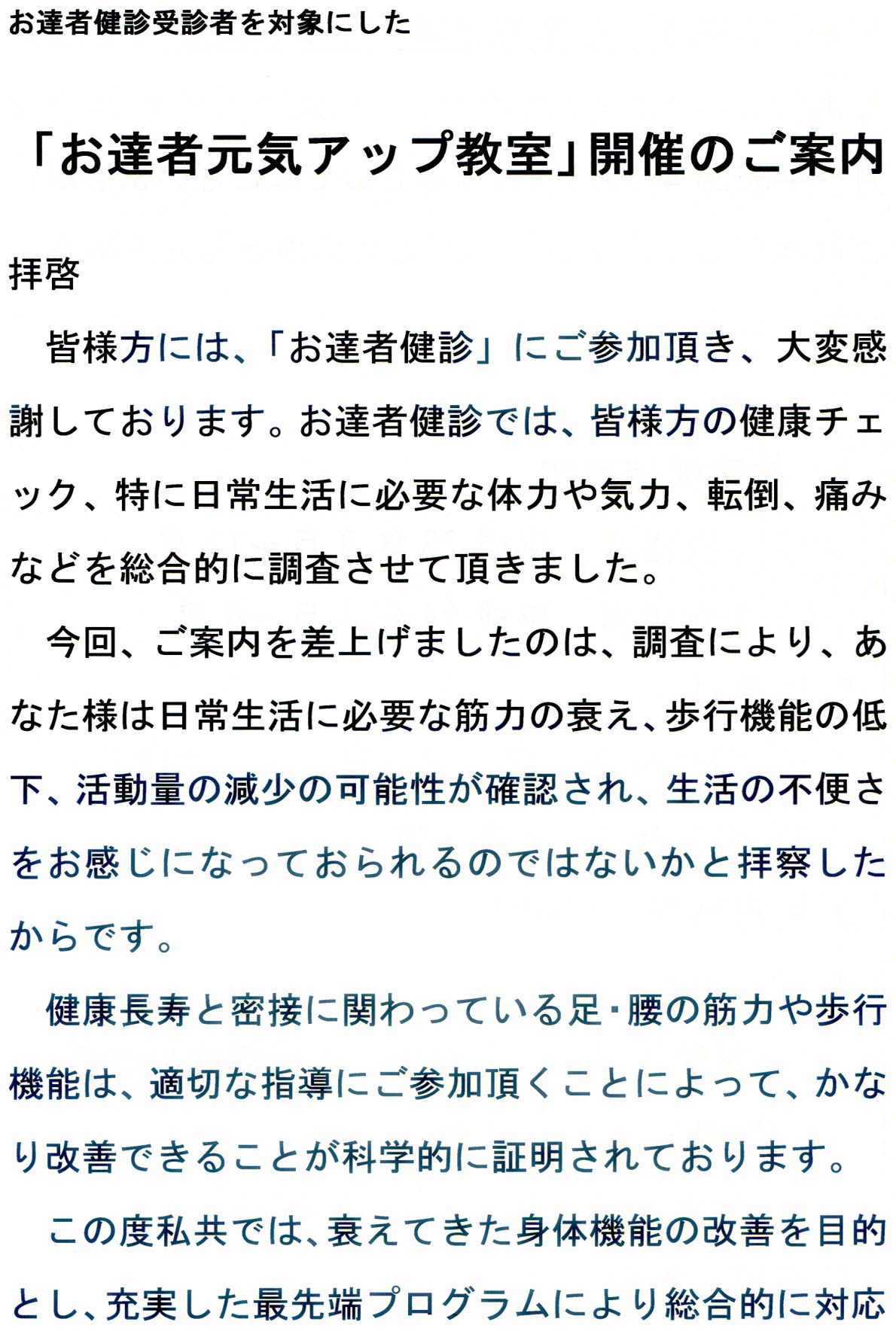


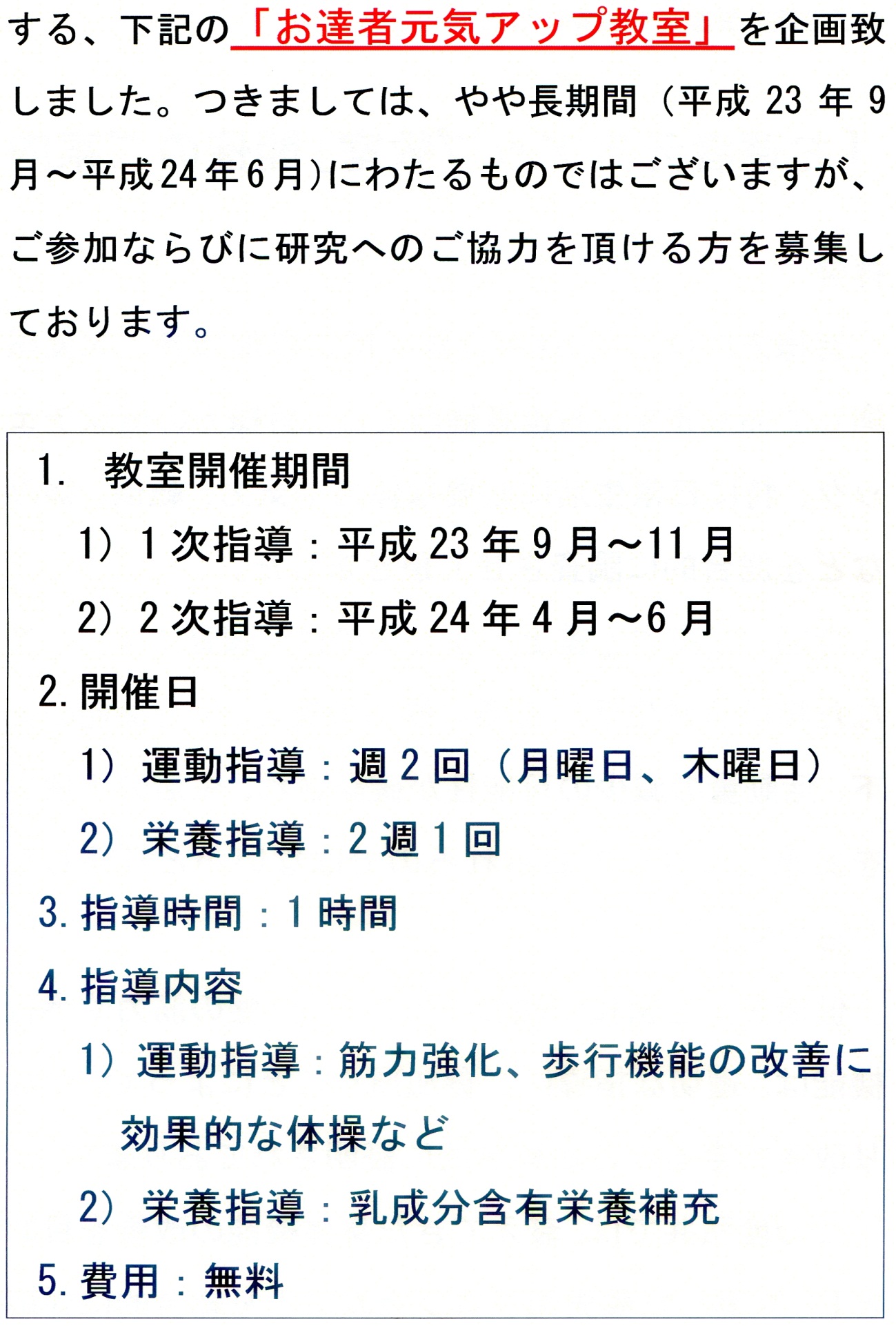


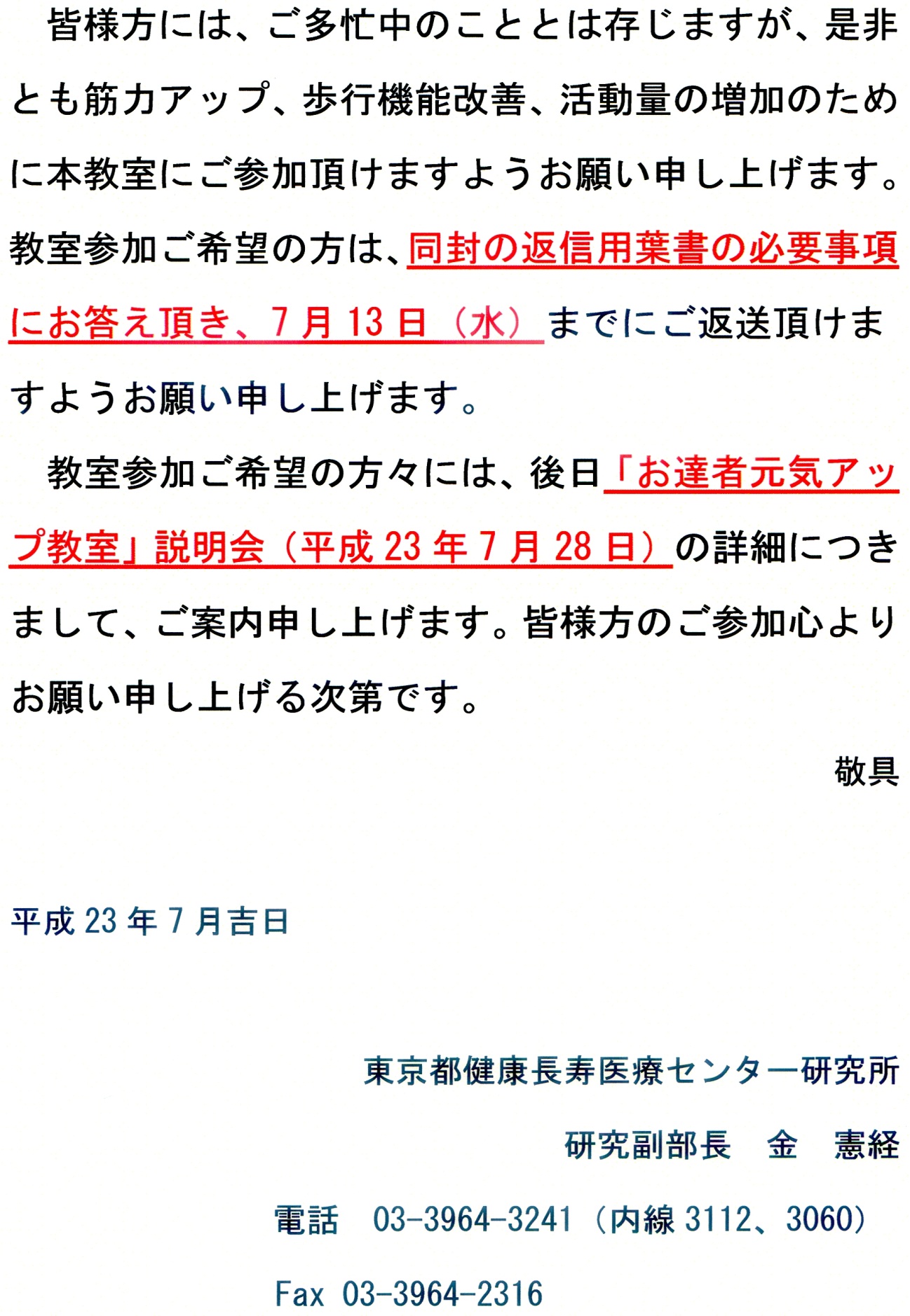


Information Session Documents

Informed Consent Form
